# Effect of Selectively Introducing Arginine and D-Amino Acids on the Antimicrobial Activity and Salt Sensitivity in Analogs of Human Beta-Defensins

**DOI:** 10.1371/journal.pone.0077031

**Published:** 2013-09-27

**Authors:** Sudar Olli, Nandini Rangaraj, Ramakrishnan Nagaraj

**Affiliations:** CSIR-Centre for Cellular and Molecular Biology, Hyderabad, India; Indian Institute of Science, India

## Abstract

We have examined the antimicrobial activity of C-terminal analogs of human β-defensins HBD-1and-3 wherein lysines have been selectively replaced by L- and D-arginines and L-isoleucine substituted with its D-enantiomer. The analogs exhibited antibacterial and antifungal activities. Physiological concentration of NaCl did not attenuate the activity of the peptides against Gram-negative bacteria considerably, while some attenuation of activity was observed against *S. aureus*. Variable attenuation of activity was observed in the presence of Ca^2+^ and Mg^2+^. Introduction of D-amino acids abrogated the need for a disulfide bridge for exhibiting activity. Confocal images of carboxyfluorescein (CF) labeled peptides indicated initial localization on the membrane and subsequent translocation into the cell. Analogs corresponding to cationic rich segments of human defensins substituted with L- and D-arginine, could be attractive candidates for development as future therapeutic drugs.

## Introduction

Mammalian defensins are cationic peptides with three disulfide bridges [1-3]. They are important components of the host innate immune defense system [3,4]. Based on how the six cysteines are linked to form the three disulfide bonds, mammalian defensins are classified as α- and β-defensins [1,2]. The β-defensins are found in epithelial cells that line mucosal surfaces [5-7] which provide the first line of defense between an organism and the environment. To date, four human β-defensins (HBD-1 to -4) have been characterized [8-14].

HBD-1 exhibits antibacterial activity at micromolar concentrations against Gram-negative bacteria, but its activity is attenuated in the presence of NaCl [15]. When tested *in vitro*, HBD-1 is relatively less potent against the Gram-positive bacterium *Streptococcus* *aureus* [9,15,16]. HBD-2 exhibits considerable activity against Gram-negative bacteria, but is bacteriostatic against *S. aureus* [17]. Its antibacterial activity against Gram-negative bacteria is strongly attenuated in the presence of high salt [17,18]. Activity against *S. aureus* is not sensitive to the presence of high salt concentration. HBD-3 exhibits broad spectrum antibacterial activity that is not attenuated at low salt concentration [10].

Several studies have indicated that all the three disulfide bridges are not required for exhibiting antimicrobial activity in HBD-1-3 [19-29]. The effect of substituting L- by D-amino acids in α- and β-defensins has been examined. The α-defensins HNP-1, HD-5 and the β-defensin HBD-2 and their D-enantiomers exhibited identical activity against *Escherichia. coli* [30]. However, against *S. aureus* the D-enantiomers of HNP-1 and HD-5 showed reduced antibacterial activity. The L- and D-enantiomers of HNP-4 showed comparable activity against *S. aureus* and *E. coli* [30]. Substitution of single L- amino acids by D-enantiomers in the β-bulge region of HNP-2 resulted in varying activity, but complete loss in activity was not observed [31]. The favourable biophysical properties of R as compared to K have been discussed extensively to understand the cationicity of human defensins, particularly in α-defensins, with respect to their biological activities [31-33]. Zou et al., have observed that in α-defensins, R is a better residue as compared to K with respect to their ability to kill bacteria [34]. They have rationalized their observations based on differences in the physico-chemical properties between R and K. In HBD-1, the K→R change did not result in markedly improved antibacterial activity as compared to the parent HBD-1 [34]. This could arise due to the distribution of R residues in the two defensins. In HNP-1, R residues are distributed throughout the sequence whereas in HBD-1, they are clustered in the C-terminal region. Investigations on structure-activity relationship in mouse paneth cell α-defensin Crp-4 [32,33] suggest that high R content may favour improved antimicrobial activity under physiological conditions. In HBD-1 (between the third cysteine and the C-terminal amino acid) there are four K and one R residues. HBD-3 has five K and four R residues in the same region, apart from two E residues. Despite these differences, peptides spanning the cationic C-terminal region of HBD-1-3, constrained by a single disulfide bridge show comparable antibacterial activity [23]. Since substitution of K by R in the cation rich segment could conceivably lead to improved antimicrobial properties, we have investigated the effect of increasing the number of R residues in a peptide corresponding to the C-terminal segment of HBD-1. In order to examine whether orientation of side-chain residues in the C-terminal segments of HBD-1and -3 would modulate their antimicrobial activity, the effect of introduction of D-amino acids, R and I in the place of their L-enantiomers on antimicrobial activity, was also investigated.

## Materials and Methods

### Reagents

9-Fluorenylmethoxycarbonyl (Fmoc) protected amino acids were purchased from Novabiochem (La Jolla, CA). Fmoc-L-arginine 4-hydroxymethylphenoxy acetic acid-polyethylene glycol-polystyrene (PAC-PEG-PS) resins were obtained from Millipore (USA). N-Hydroxybenzotriazole hydrate (HOBT) and 2-(1H-benzotriazole-1-yl)-1,1,3,3-tetramethyluronium hexaﬂuorophosphate (HBTU) were from Advanced Chemtech (Louisville, KY). Piperidine was from Loba-Chemie Pvt. Ltd (India). Reagents for deprotection of peptides were purchased from Sigma Chemical Co. (St. Louis, MO).

### Peptide synthesis

Peptides were synthesized by solid-phase methods manually, using Fmoc-L-arginine-4-(hydroxymethyl) phenoxyacetamidomethyl resin and 9-ﬂuorenylmethoxy carbonyl chemistry as described earlier [23]. Peptides were cleaved from the resin using triﬂuoroacetic acid containing thioanisole, meta-cresol and ethanedithiol (10:1:1:0.5, v/v). Formation of disulﬁde bonds was accomplished by air oxidation in 20% (v/v) aqueous dimethyl sulfoxide [35] at a concentration of 0.5 mg/ml for 24 h at room temperature. Peptides were puriﬁed by HPLC on a reversed phase C-18 (Hi-pore reversed phase column 4.6 mm 250 mm) column using gradients of solvents: A; 0.1% (v/v) TFA in H_2_O, B; 0.1% (v/v) TFA in CH_3_CN. Puriﬁed peptides were characterized by Matrix-assisted laser desorption ionization time-of-ﬂight mass spectrometry on a ABI Voyager DE STR MALDI-TOF mass spectrometer (Perseptive Biosystems) in the Proteomics Facility of CSIR-CCMB using recrystallized α–cyano-4-hydroxycinnamic acid as matrix.

Labeling of peptides with carboxyﬂuorescein (CF) at the free amino group of the N-terminal amino acid was carried out by treating 10 mg of resin-bound peptide with 0.8 ml of dimethylformamide containing CF and activating agents as described earlier [36]. The deprotection of CF-labeled peptides from the resin, puriﬁcation, and characterization by mass spectrometry were carried out as described earlier [23].

### Antibacterial activity

Bacterial strains used were *E. coli* (MG 1655), *S. aureus* (ATCC 8530), and *P. aeruginosa* (NCTC 6751). The antibacterial activity of the peptides was examined in sterile 96 well plates at a ﬁnal volume of 100 µl as follows: Bacteria were grown in nutrient broth (Bacto Difco nutrient broth) to mid-log phase and diluted to 10^6^ colony forming units (cfu)/ml in 10 mM sodium phosphate buffer (pH 7.4). Bacteria were incubated with different concentration of peptides for 2 h at 37°C and suitably diluted aliquots were spread on nutrient agar plates. After the plates were incubated at 37°C for 18 h, colonies formed were counted. Lethal concentration (LC) is the concentration of the peptides at which no viable colonies were formed. Cell survival is expressed as a percentage of the control. Percentage killing was calculated as: [(colonies from control cells- colonies from treated cells)/colonies from control cells x 100]. The LC determined was average of three independent experiments done in duplicate. In control experiments, cells were incubated with only buffer.

Activity of the peptide analogs were also tested in the presence of 1mM DTT. The disulfide bridges were broken by incubation with DTT at 37°C for 1h. Antimicrobial assay was performed with reduced peptide in the presence of 1 mM DTT. In all the experiments, untreated peptide and cells in the presence of 1mM DTT were used as controls.

To determine the effect of salt on antibacterial activity, different concentrations of NaCl was included in the incubation buffer at their LC. Different concentrations of divalent cations Ca^2+^ and Mg^2+^ (as their chloride salts), were included in the buffer to determine their effect on activity at lethal concentration (LC) of the peptides. In control experiments, cells were incubated with only buffer. Data are presented as mean ± standard deviation computed for three independent replicates.

### Candidacidal activity

Minimum fungicidal concentrations (MFC) of the peptides were determined by growing *C. albicans* aerobically in yeast extract-peptone-dextrose (YEPD) medium at 30°C. After 20 h, 0.5 ml from this suspension was subcultured for 2 h in 20 ml of YEPD broth to obtain a mid-log-phase culture. Cells were harvested by centrifugation, washed with 10 mM phosphate buffer (PB), pH 7.4, and resuspended in the same buffer, and the concentration was adjusted to 10^6^ cells/ml. Aliquots of diluted cells were incubated with peptides in 100 µl volume at 30°C for 2 h. Cell suspensions were diluted and spread on YEPD agar plates and the plates were incubated for 24 h at 30°C. Colonies were counted, and the concentrations of the peptides at which no viable colonies were formed were taken as the Minimum fungicidal concentration (MFC). Cell survival was expressed as a percentage of the control. Percentage killing was calculated as [(colonies from control cells- colonies from treated cells)/colonies from control cells x 100].

The average of results from three independent experiments done with duplicate samples was taken for the calculation of MFC. In control experiments, cells were incubated with only buffer.

### Circular dichroism (CD)

Spectra were recorded in 10 mM phosphate buffer (pH 7.4), TFE, and 10 mM SDS micelles on a JASCO J-715 automatic recording spectropolarimeter at 25°C using a quartz cell of 1 mm path length. Each spectrum (185-250 nm) was an average of six scans. Data are represented as mean residue ellipticities.

### Confocal microscopy

Localization of CF-labeled peptides was examined by treating *E. coli* and *C. albicans* with CF-peptides and FM4-64 and propidium iodide (PI), respectively. *E. coli* and *C. albicans* were grown overnight in nutrient broth and yeast extract-peptone-dextrose medium, respectively, washed with 10 mM phosphate buffer (pH-7.4) and the concentration were adjusted to 1 x 10^7^ cells/ml in 10 mM phosphate buffer. These cells were treated with sub-lethal concentration of peptide for different time intervals. Peptides were incubated for 5 min, 10 min, 20 min and 30 min at 37°C or 30°C for *E. coli* and *C. albicans* respectively, to capture different stages of peptide entry, localization on the membrane and killing. After addition of peptide, the live cells were examined with a Zeiss LSM 510 META confocal microscope. Optical sectioning was done at 1 airy unit by using the 488- and 543-nm-wavelength laser lines with a 63 water lens objective. Emission data were collected using 500- to 530-nm band-pass and 565- to 615-nm band-pass ﬁlters for CF and PI, respectively, in the multitrack mode. Z-sections were acquired at 0.35-µm intervals and projected using the LSM-FCS software version 3.2. The bright-ﬁeld images were obtained simultaneously using the transmitted-light detector. Three independent experiments were done in case of bacteria and fungi to record different stages of peptide entry into the cell. Images shown are representative of the different events (Initial interaction with the membrane and subsequent translocation into the cells) that could be captured from these independent experiments.

## Results

The sequences of HBD-1and-3 and the peptides investigated in the present study are shown in Table 1. In HC-1(R), two K residues at the C-terminal end were replaced by R resulting in a R/K ratio of 3:2 as compared to 1:4 in Phd-1. Two R and I residues were substituted with their D-enantiomers in [D]HC-1(R) so that the D-amino acids are distributed across the sequence from N-to C-terminus. Phd-3 has a R/K ratio of 4:5. Since this peptide was rich in R, analog [D]HC-3(R) was generated where the C-terminal K residue was substituted with R and D-enantiomers of R and I were introduced along the peptide sequence. All the peptides have a R/K ratio > 1.

**Table 1:** Primary structure of HBD-1 and-3 and their analogs.

**Peptide**	**Sequence^a^**
HBD-1	DHYN**C^1^**VSSGGQ**C^2^**LYSA**C^3^**PIFTKIQGT**C^2^**YRGKAK**C^1^C^3^**K
Phd-1^b^	A **C^1^** P I F T K I Q G T Y R G K A K **C^1^** K
HC-1(R)	A **C^1^** P I F T K I Q G T Y R G R A K **C^1^** R
[D]HC-1(R)	A **C^1^** P ^D^I F T K ^D^I Q G T Y ^D^R G ^D^R A K **C^1^** R
HBD-3	GIINTLQKYY**C^1^**RVRGGR**C^2^**AVLS**C^3^**LPKEEQIGK**C^2^**STRGRK**C^1^C^3^**RRKK
Phd-3^b^	S **C^1^** L P K E E Q I G K S T R G R K **C^1^** R R K K
[D]HC-3(R)	S **C^1^** L P K E E Q ^D^I G K S T ^D^R G ^D^R K **C^1^** ^ D^R ^D^R K R

^a^ Superscript numbers adjacent to cysteines in bold indicate disulfide connectivity. In [D]HC-1,-3(R), D-amino acids are represented by D superscript. ^b^ Sequences are from [23].

The antimicrobial activity of the peptides is summarized in Table 2. All the peptides exhibit activity against Gram-negative, Gram-positive bacteria as well as *C. albicans*. The activity of HBD-1 analogs, HC-1(R) and [D]HC-1(R) are comparable except against *C. albicans*, where HC-1(R) is more active. The analog of HBD-3 shows comparable activity against bacteria and *C. albicans*. [D]HC-(3) is 2-fold more active against bacteria as compared to HC-1(R) and [D]HC-1(R). Loss in antimicrobial activity was observed for HC-1(R) when the disulfide bridge was broken by DTT similar to Phd-1-3. The activities of the analogs with D-amino acids, [D]HC-1(R) and [D]HC-3(R), were not lost when the disulfide bridge was broken by DTT. All the analogs were more active than Phd-1 and -3.

**Table 2:** Antimicrobial activity of HBD-1 and-3 analogs.

	**LC (µM)**			**MFC (µM)**
	*E. coli*	*P. aeruginosa*	*S. aureus*	*C. albicans*
**Phd-1^a^**	**15**	**-**	**20**	**18**
**Phd-3^a^**	**17**	**-**	**17**	**16**
**HC-1(R)**	**5**	**5**	**5**	**2.5**
**[D]HC-1(R)**	**5**	**5**	**5**	**5**
**[D]HC-3(R)**	**2.5**	**2.5**	**2.5**	**2.5**

The values reported are average of three independent experiments done in duplicates. Variations observed were <5%. ^a^ Values represented are from [23],- denotes not determined.

**Figure 1:**
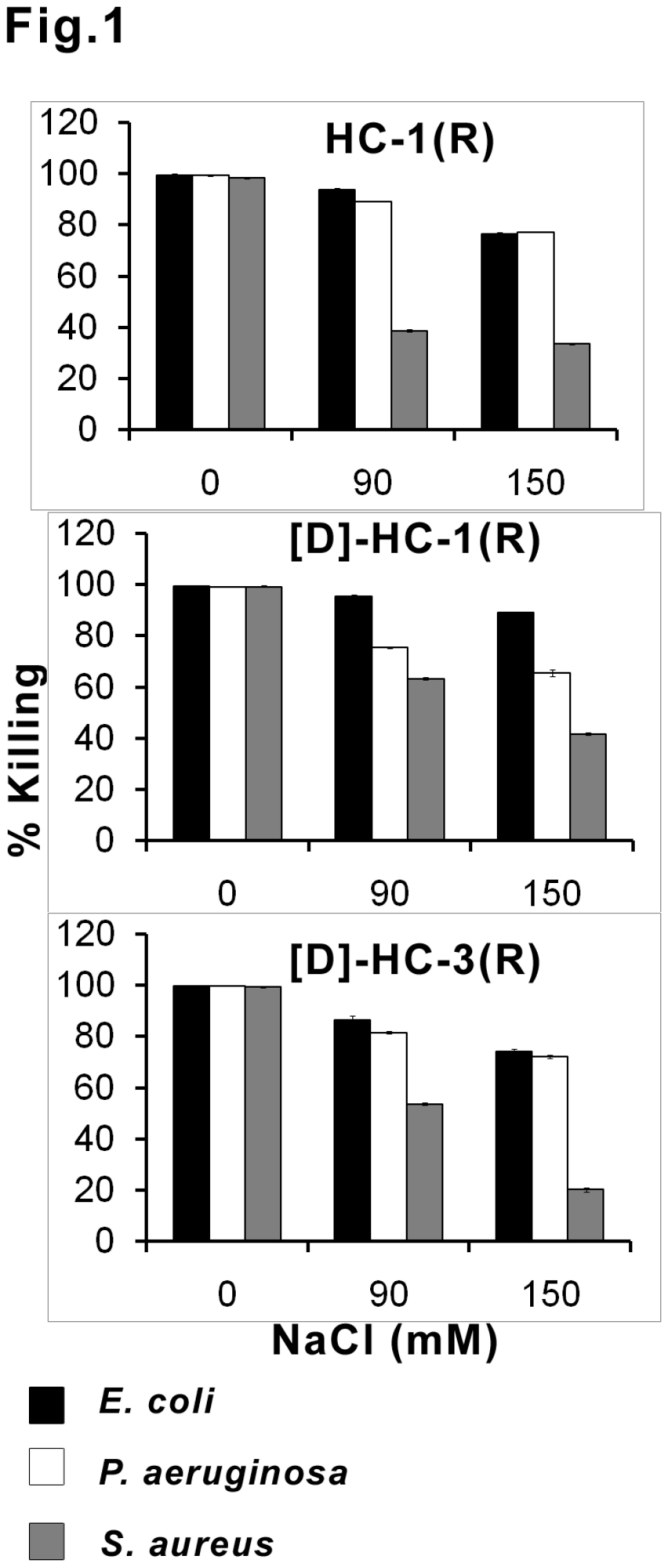
Effect of NaCl on antibacterial activity. Mid-log phase bacteria (10^6^ CFU/mL) were incubated with peptides at their LC in the absence and presence of the indicated concentrations of NaCl. The data are mean values of three independent experiments and the error bars represent standard deviation of the measurements. Standard deviation values ranged between 0.3-1.5.

**Figure 2:**
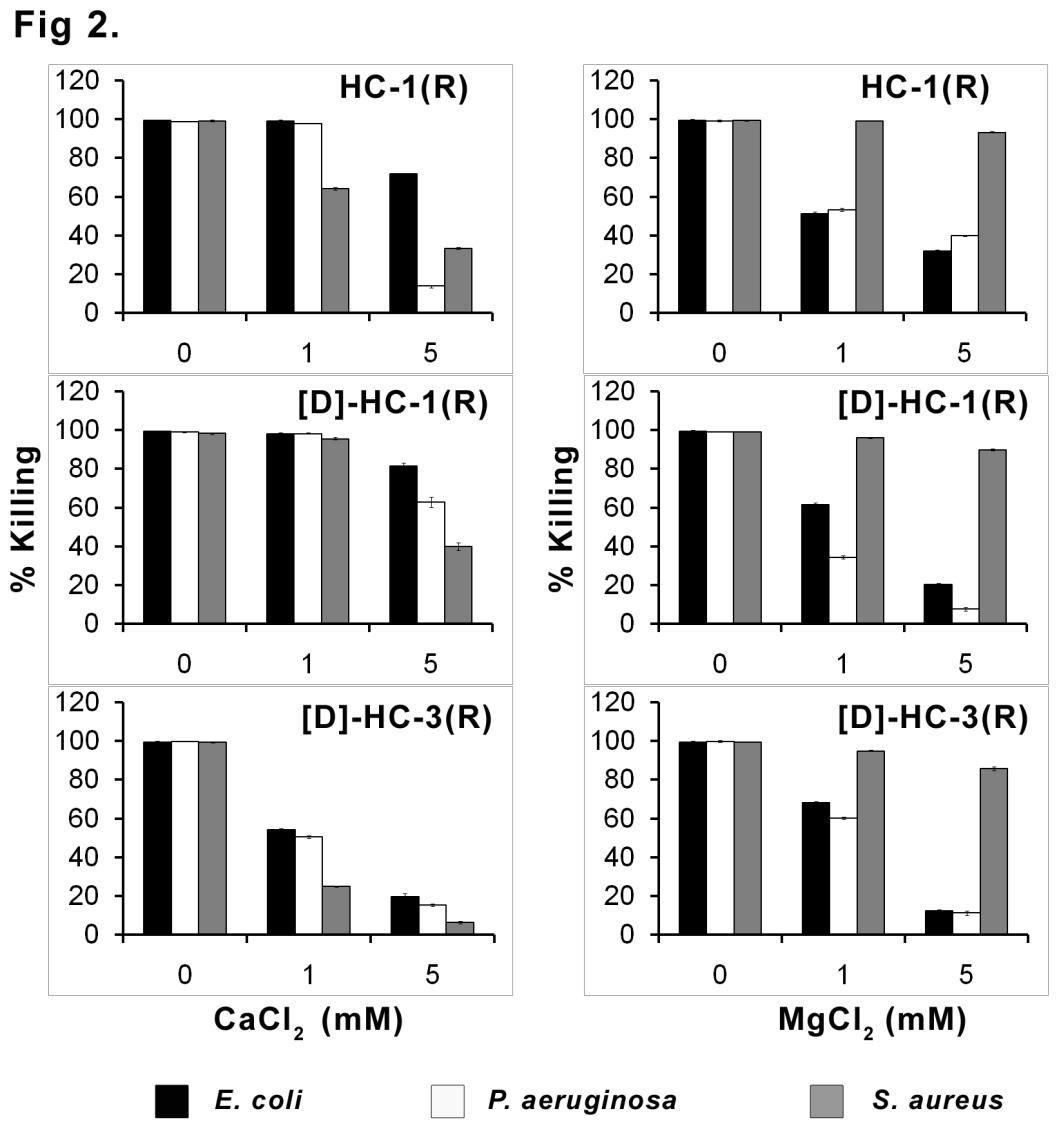
Effect of divalent cations on antibacterial activity. Mid-log phase bacteria (10^6^ CFU/mL) were incubated with peptides at their LC in the absence or presence of the indicated concentrations of CaCl_2_ and MgCl_2_. The data are mean values of three independent experiments and the error bars represent standard deviation of the measurements. Standard deviation values ranged between 0.2-2.7.

The antibacterial activity of the peptides in the presence of varying concentration of NaCl is shown in Figure 1. Even at 150 mM, considerable activity was observed against Gram-negative bacteria. The antibacterial activity against *S. aureus* was attenuated to a greater extent. The effect of Ca^2+^ and Mg^2+^ on antibacterial activity is shown in Figure 2. In the presence of 1 mM Ca^2+^, the activity of HC-1(R) and [D]HC-1(R) is not attenuated against Gram-negative bacteria, whereas, loss in activity is observed for [D]HC-3(R) that is further attenuated at 5 mM Ca^2+^. At 5 mM Ca^2+^, loss in activity is more pronounced for HC-1(R) and [D]HC-3(R) against *P. aeruginosa* as compared to [D]HC-1(R). Loss in activity is observed for HC-1(R) against *S. aureus* but not for [D]HC-1(R) at 1 mM Ca^2+^. However, loss in activity for all the peptides is observed at 5 mM Ca^2+^. Loss in activity is evident for all the peptides in the presence of Mg^2+^against Gram-negative bacteria but not *S. aureus*. Loss in activity at 5 mM Mg^2+^ against *P. aeruginosa* is more for [D]HC-1(R) and [D]HC-3(R) as compared to HC-1(R).

**Figure 3:**
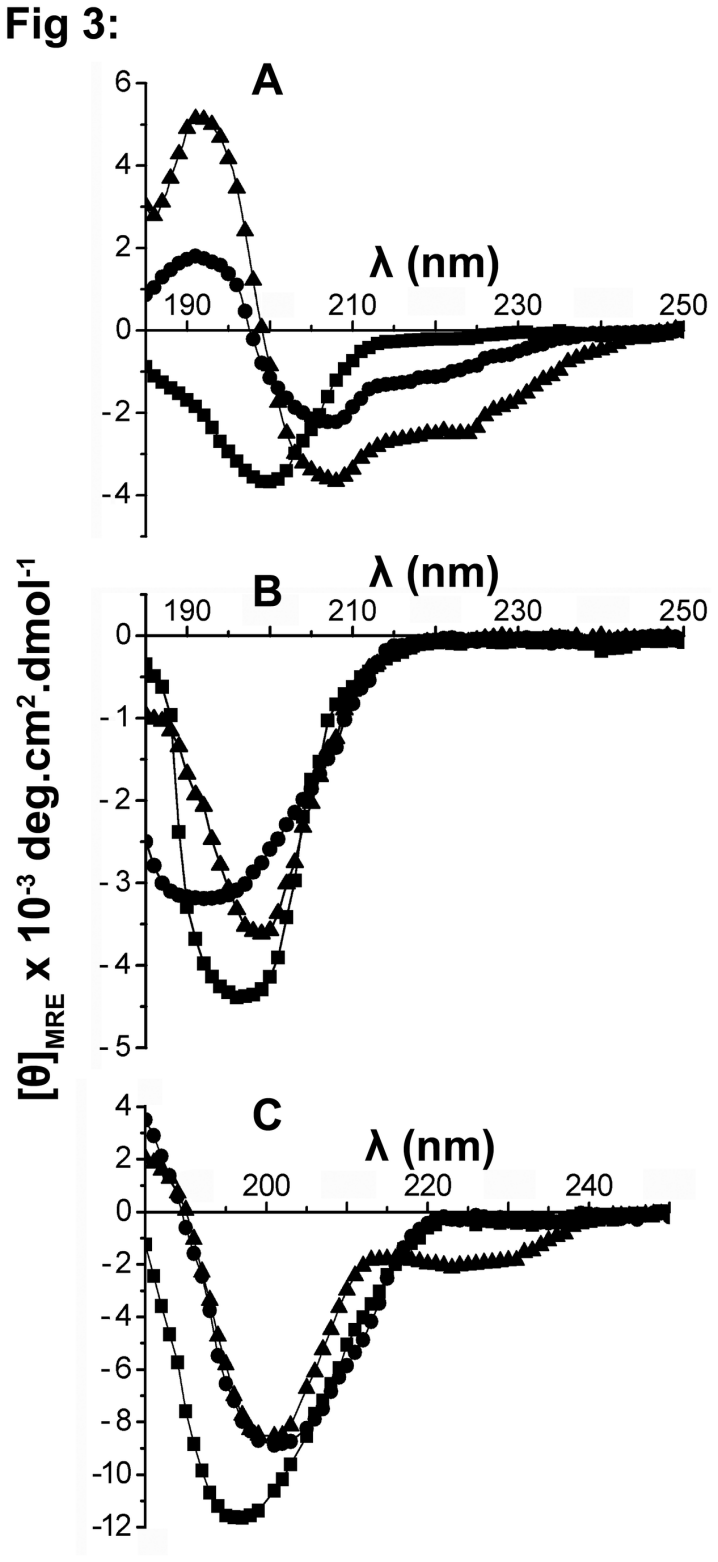
Circular dichroism spectra of peptides. (A) HC-1(R), (B) [D]HC-1(R) , (C) [D]HC-3(R). CD spectra were recorded in 10 mM phosphate buffer (■), 10 mM SDS (●) and TFE (▲).

Secondary structure of the peptides was examined in buffer, SDS micelles and TFE (Figure 3). In buffer, HC-1(R) shows a minimum ~200 nm with cross-over < 190 nm indicating largely unordered conformation. In SDS micelles, a minimum ~210 nm is observed with cross over at 200nm suggesting population of β-structure. The TFE spectrum is characterized by a positive band ~190 nm, a negative band ~208 nm with a shoulder at 220 nm characteristic of helical conformation. [D]HC-1(R) shows a single minimum ~200 nm with cross-over < 190 nm in all the solvents indicating conformational flexibility and largely unordered conformation. [D]HC-3(R) shows similar negative band ~200 nm with a cross-over ~190 nm in all the solvents. The spectra suggest a fraction of peptide populating β-conformation that is independent of solvent unlike the other peptides.

**Figure 4:**
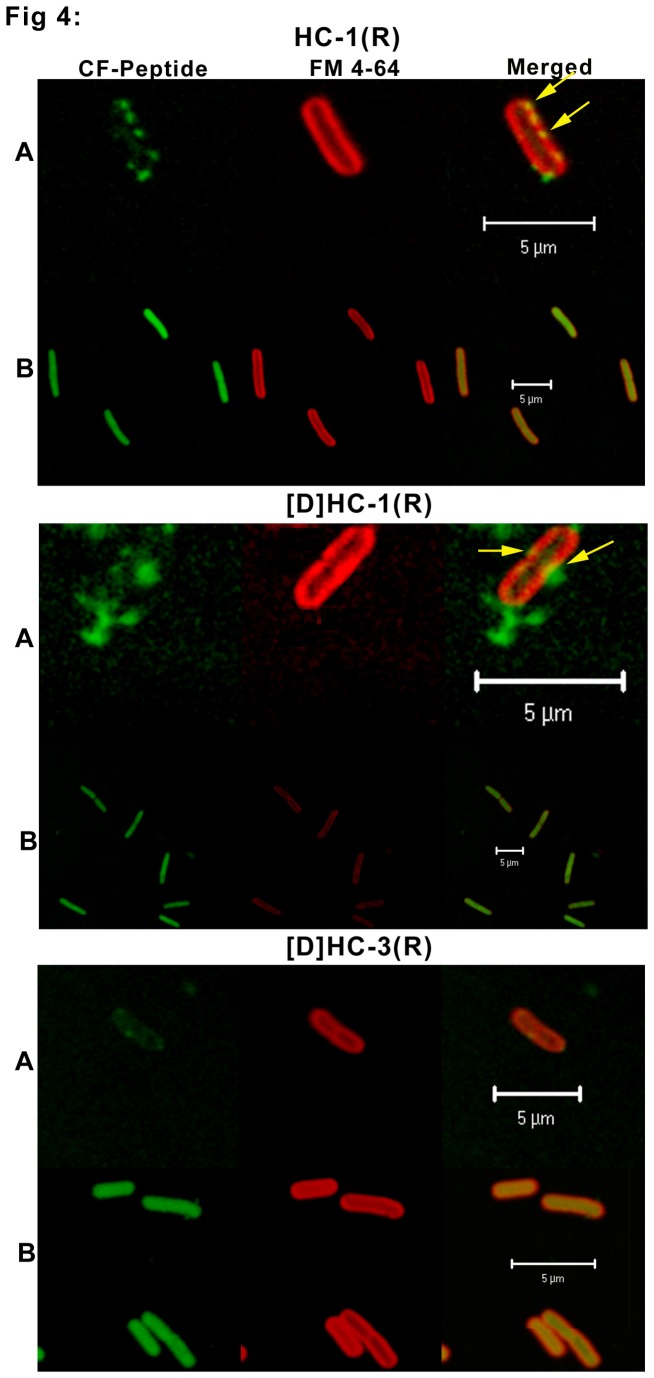
Confocal microscope images of *E. coli* in the presence of CF-labeled HC-1(R), [D]HC-1(R) and [D]HC-3(R). Bacterial cells (1 x 10^7^) were treated with CF labeled peptide and FM4-64 and were incubated for different time points. Panel A represents an early time point where the peptide interacts with the bacterial membrane. Panel B represents a late time point wherein the peptide is found completely diffused inside the cell. The bar represents 5 µm.

Interaction of peptides with *E. coli* and *C. albicans* was examined using CF-labeled peptides by confocal microscopy. Antimicrobial activity of the CF-labeled peptides was comparable to that of the unlabeled peptides. CF labeled HC-1(R) and [D]-HC-1(R) interacted with *E. coli* (Figure 4) in a similar manner. The peptides were initially localized on the inner membrane (Panel A). The peptides are observed on the bacterial membrane at only few points, and these points of entry of peptide over the membrane is seen as a yellow patch (as indicated by the arrows in Panel A). Panel B shows localization within the bacteria without disruption of the inner membrane. As the peptide enters the cells rapidly, peptide localization on the bacterial membrane was not captured for a group of bacteria. Instead the ones shown in the figure are representative of a few that could be captured. Peptide [D]-HC-3(R) appears to enter the cells rapidly. Localization on the membrane surface could not be captured as in the case of CF labeled HC-1(R) and [D]-HC-1(R). Loss of inner membrane intergrity is not discernible.

**Figure 5:**
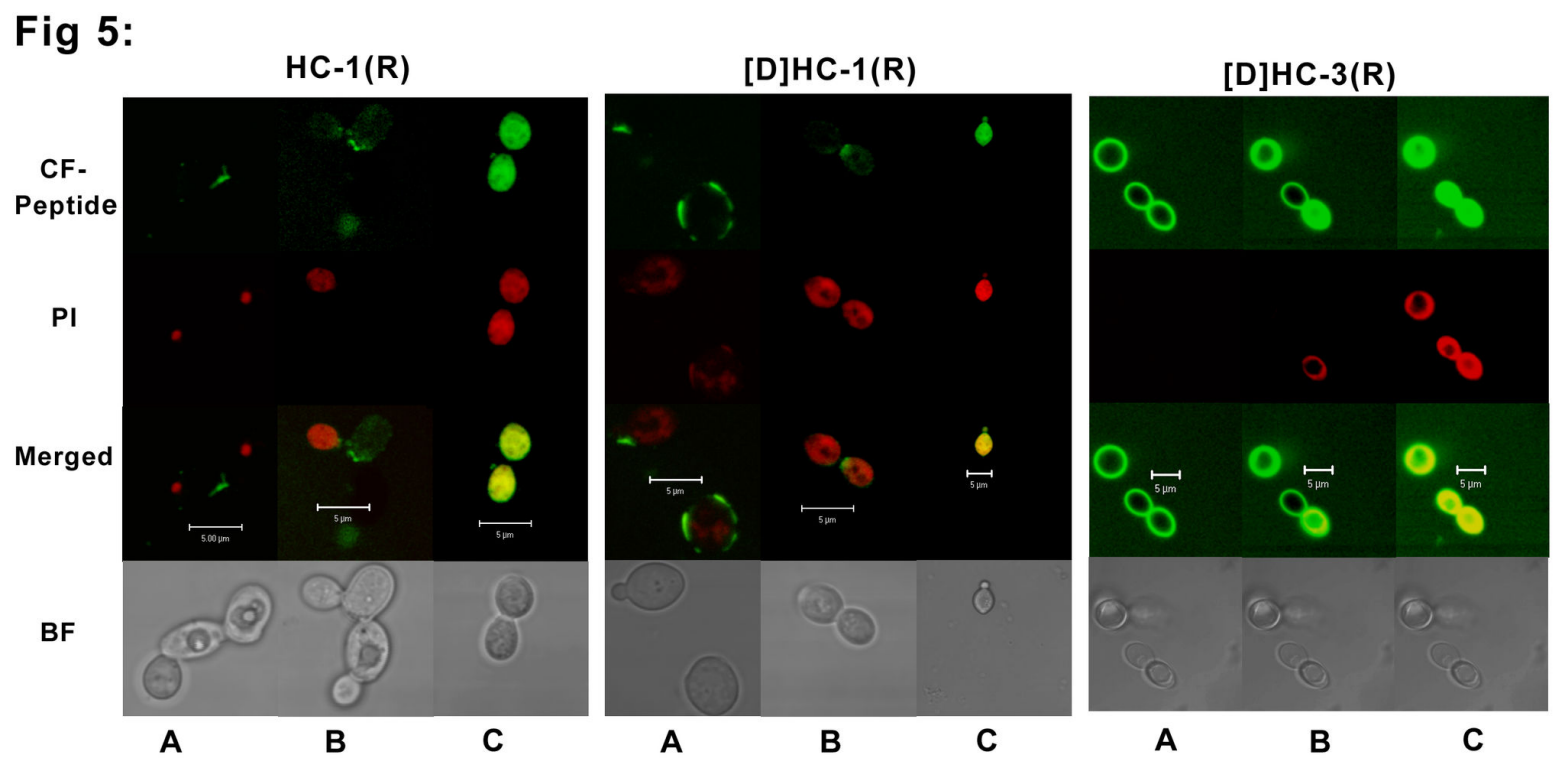
Confocal microscope images of *C. albicans* in the presence of CF-labeled HC-1(R), [D]HC-1(R) and [D]HC-3(R). *C. albicans* cells (1 x 10^7^) were treated with CF labeled peptide and 2 µg/ml of PI. The treated cells were incubated for different time points to capture different events during the process of peptide entry into the cell. Panel A represents an early time point wherein the peptide is found over the membrane. Panel B represents the stage where, the peptide diffuses into the cells (green) and the dying cells are taking up PI stain (red) Panel C represents dead cells (yellow in the merged panel) showing the diffused peptide (green) and the diffused nuclear material (red). The bar represents 5 µm.

The interaction of peptides with *C. albicans* is shown in Figure 5. In case of HC-1(R) and [D]-HC-1(R), peptides are seen on the membrane as patches at a few points, whereas in case of [D]-HC-3(R), the peptide is seen distributed uniformly all over the membrane as a ring (Panel A). Subsequently, all the three peptides translocate inside the cell and the killing process is initiated, as indicated by PI uptake by the dying cells (Panel B). In the dead cells, completely diffused nuclear material (red) and uniformly diffused peptide (green) are seen as yellow in the merged panel (Panel C).

## Discussion

Human defensins are endogenous host-defense antimicrobial peptides with potent antibacterial activity against various microorganisms [2,3,6] and therefore could be attractive candidates for the development as therapeutic agents. However, their size, complexity of disulfide pairing and attenuation of activity in the presence of high NaCl concentration [3,15,16,18,37-39] is likely to come in the way of their development as therapeutic agents. Studies on hybrid and cyclic analogs of defensins have shown promise for engineering novel, salt-insensitive antimicrobial agents [40,41]. Of the β-defensins, HBD-3 has been of particular interest as it appears to possess better broad-spectrum antimicrobial activity as compared to HBD-1 and HBD-2 [10,17,18,41]. It has been demonstrated that the analogs of HBD-1-3: Phd-1, Phd-2 and Phd-3 respectively, corresponding to the C-terminal region of the parent peptides having a single disulfide bridge, displayed activity against both Gram-negative and Gram-positive bacteria and *C. albicans* [23,29]. We have explored the effect of substituting K with R so that the R/K ratio is >1. D-isomers of R and I were introduced to generate diastereomeric peptides.

The substitutions K to R and introduction of D-amino acids in peptides spanning the C-terminal segment of HBD-1, -3 resulted in marginally enhanced activity as compared to Phd-1,-3 [23]. However, unlike in the case of Phd-1,-3 [23], the activity of the analogs in the present study retained considerable activity at high concentrations of NaCl. The analogs containing D-amino acid were active even in the reduced form. Hence, K→R change where R is in D-form abrogates the requirement of disulfide bridge for activity.

Zou and coworkers have shown that K→R change modulates the activity of α-defensins to a greater extent as compared to β-defensin [34]. Infact, the K→R change has only a marginal effect on the activity of HBD-1. Our results indicate that the K→R change results in considerable activity at high NaCl concentrations in the peptide corresponding to the C-terminal fragment of HBD-1. The greater potency of HC-1(R) and the [D] HC-1(R) could arise due to more effective interaction with the negatively charged bacterial cell surface. This observation can be attributed to the presence of R in place of K as R has a stronger ability to engage in electrostatic interactions such as salt bridges, H-bonds and cationic-aromatic and cationic-π contacts [34]. Also, the observation that divalent cations attenuate the activity only at 5 mM concentration also supports our argument.

In the present study, confocal images clearly showed that the peptides did not cause gross membrane disruption and membrane destabilization. The images indicate that HC-1(R) and [D]-HC-1(R) were localized on the membranes initially and subsequently translocated across the membrane. Localization of [D]-HC-3(R) on the membrane was not discernible. The peptide appears to rapidly enter bacterial cells. The mode of entry of HC-1(R) and [D]-HC-1(R) into *C. albicans* is different from [D]-HC-3(R). After entry, it is likely that the mechanism of killing is similar.

Electrostatic charge-based mechanisms rather than formation of bilayer-spanning pores have been proposed for β-defensins [41-49]. The fact that the antimicrobial activity of the peptides is salt-resistant, suggests that electrostatic interaction is crucial for membrane permeability. As arginines have a stronger ability to engage in electrostatic interactions, and are probably less sufficiently screened by salts to diminish the binding to the negatively charged lipid components in membranes, our findings appear consistent with the proposed electrostatic charge-based mechanisms for the mode of action. The high concentration of cationic defensin peptides in the cytoplasm probably interferes with metabolic activities resulting in rapid cell death as proposed by Hancock and co-workers [50,51].

Components of bacterial membranes appear to play an important role in discriminating interactions with different defensins. These components include lipopolysaccharides [52,53], teichoic acids [54], glycosaminoglycans and cell wall precursor lipid II [55]. All the peptides in the present study exhibited potent activity against both Gram-positive and Gram-negative bacteria as well as *C. albicans*, which indicates that the peptides, though are conformationally flexible, have the ability to interact with membranes with varying lipid composition.

In summary, our study indicates that substituting R for K and introducing D-amino acids results in peptides whose antibacterial activity is attenuated only marginally at high NaCl concentrations. When D-amino acids are introduced, even the linearized peptides are active unlike their counterparts with L-amino acids. Selective introduction of D-amino acids would result in increased structural flexibility which appears to result in improved antimicrobial activity. While it remains to be established that similar engineering of full length defensins would also result in improved antimicrobial activity, short peptides with broad spectrum antimicrobial activity can be engineered from defensins, as described in this study. Such peptides could be attractive candidates for the development of therapeutic agents.
